# GXD’s RNA-Seq and Microarray Experiment Search: using curated metadata to reliably find mouse expression studies of interest

**DOI:** 10.1093/database/baaa002

**Published:** 2020-03-04

**Authors:** Constance M Smith, James A Kadin, Richard M Baldarelli, Jonathan S Beal, Olin Blodgett, Sharon C Giannatto, Joel E Richardson, Martin Ringwald

**Affiliations:** The Jackson Laboratory 600 Main Street Bar Harbor, ME 04609, USA

## Abstract

The Gene Expression Database (GXD), an extensive community resource of curated expression information for the mouse, has developed an RNA-Seq and Microarray Experiment Search (http://www.informatics.jax.org/gxd/htexp_index). This tool allows users to quickly and reliably find specific experiments in ArrayExpress and the Gene Expression Omnibus (GEO) that study endogenous gene expression in wild-type and mutant mice. Standardized metadata annotations, curated by GXD, allow users to specify the anatomical structure, developmental stage, mutated gene, strain and sex of samples of interest, as well as the study type and key parameters of the experiment. These searches, powered by controlled vocabularies and ontologies, can be combined with free text searching of experiment titles and descriptions. Search result summaries include link-outs to ArrayExpress and GEO, providing easy access to the expression data itself. Links to the PubMed entries for accompanying publications are also included. More information about this tool and GXD can be found at the GXD home page (http://www.informatics.jax.org/expression.shtml).

**Database URL:** http://www.informatics.jax.org/expression.shtml

## Introduction

The mouse Gene Expression Database (GXD) is an easily searchable database that is freely available online for use by researchers to accelerate progress toward understanding the molecular basis of development and disease (1–3). To accomplish this GXD collects data reporting endogenous gene expression patterns in wild-type (WT) and mutant mice. These data are integrated with all the other genetic, functional, phenotypic and disease-related information in the larger Mouse Genome Informatics resource (MGI; 4), of which GXD is an integral part. This makes GXD’s expression data widely accessible and amenable to many types of biologically and biomedically relevant database searches.

For over 20 years GXD’s expression data annotations have been derived from RNA *in situ* hybridization, immunohistochemistry, *in situ* reporter (knock in), reverse transcriptase-polymerase chain reaction, northern blot and western blot experiments. These data have been annotated in standardized ways, making extensive use of controlled vocabularies and ontologies. As is consistent with GXD’s focus on studies of endogenous gene expression, studies using transgenic mice (i.e. animals containing randomly inserted transgenes); animals that have been manipulated, either by substances, surgical procedures or diet regimens; cultured tissues; cell types; or cell lines are not included.

For our first foray into high-throughput expression data, GXD has sought to fulfill a critical, previously unmet community need: to enable researchers to reliably find mouse RNA-seq and microarray studies in the public repositories that are within GXD’s scope. To do this we have developed a sample and experiment metadata index and search tool for these studies. The public repositories for RNA-seq and microarray expression studies are Gene Expression Omnibus (GEO; https://www.ncbi.nlm.nih.gov/geo/; 5) and ArrayExpress (https://www.ebi.ac.uk/arrayexpress/; 6). Both resources enable data submitters to describe their data at the level of detail required to reproduce experiments, and thus be MIAME (Minimum information about a microarray experiment)- and MINSEQE (Minimum information about a high-throughput nucleotide sequencing experiment)-compliant (7). However, to facilitate data submission, completeness of data submissions and standardization of metadata are not required. In particular, GEO has chosen to provide flexibility in the data submission process, even going as far as allowing the submitters to name and define the data categories. The result is that sample and experiment metadata are largely described using free text, lacking standardization and ontological structure. For instance, our analysis revealed more than 60 differently named database fields reporting the age of samples (Table 1). Differences in term usage or spelling mean that text searches frequently fail to find experiments of interest. Further, lack of ontology usage makes hierarchical searches impossible; for example, a free text search for ‘skeletal muscle’ would not return entries studying ‘quadriceps’ or ‘soleus.’ Consequently, although ‘flexibility’ seems data submitter friendly, it exacts a heavy cost, making reliable systematic searches based on metadata impossible at these resources.

**Table 1:** Field labels used by data submitters to describe the field containing sample ‘age’ information

age	age (days old)
Age	age_days
AGE	age days postnatal
adult age	age description
age and sex	age/gender
age (day)	age group
agedays	age_group
age days	age in days
age (days)	age in months

A total of 18 field labels are included in this table. We identified at least 43 other variants.

The need for standardized, structured metadata is well recognized (cf. 8,9). Some specialized efforts have been undertaken to standardize metadata for transcriptomics experiments, combining computational tools and manual data curation. For example, Bagewadi *et al*. (10) have developed NeuroTransDB (www.scai.fraunhofer.de/NeuroTransDB.html); it contains more than 20 dimensions of curated metadata annotations for 81 studies related to neurodegenerative disease. Becnel *et al*. (11) have developed Transcriptomine (https://nursa.org/nursa/transcriptomine/index.jsf), which includes detailed metadata curation of 550 expression profiling data sets studying nuclear receptor signaling pathways.

We have pursued a broader metadata annotation effort. We have surveyed all mouse transcription profiling experiments in ArrayExpress, identified those that are within GXD’s scope and curated their sample and experiment attributes using controlled vocabularies and ontologies. These annotations are accessible via the RNA-Seq and Microarray Experiment Search (http://www.informatics.jax.org/gxd/htexp_index) that allows users to quickly and reliably find expression studies of interest in ArrayExpress and GEO, where the expression data itself can be accessed.

## Metadata Index Content

The first step in developing the GXD RNA-seq and microarray metadata index was (and is) to download high-throughput RNA expression experiment metadata from ArrayExpress. This metadata includes the experiment repository id, title and abstract and experiment type. The experiments whose metadata are downloaded are organism = Mus and experiment type = RNA-seq of coding RNA, RNA-seq of noncoding RNA, RNA-seq of coding RNA from single cells, RNA-seq of noncoding RNA from single cells, transcription profiling by array, transcription profiling by tiling array, microRNA profiling by array, microRNA profiling by high throughput sequencing or tiling path by array. The experiment type categories from ArrayExpress were mapped to the categories ‘RNA-seq’ and ‘transcription profiling by array’ at GXD.

We chose to use ArrayExpress as our repository source because at the time this project was under development, ArrayExpress was importing all experiments stored at GEO, thus ensuring our download would include information for experiments at both repositories. As of this writing, we have downloaded 16 550 experiments (Table 2); 2364 of these experiments were directly submitted to ArrayExpress and 14 186 experiments were originally submitted to GEO. Unfortunately, ArrayExpress stopped importing experiments from GEO in August 2016. Therefore, although we still download experiment metadata from ArrayExpress on a weekly basis, we are lacking the experiments most recently submitted to GEO.

**Table 2:** Data content as of 28 October 2019

16 550	Experiments downloaded from ArrayExpress
13 428	Experiments incompatible with GXD’s scope^*^
13	Experiments lacking publication required to identify allele used
3109	Experiments consistent with GXD’s scope/metadata included in index
2043	WT vs mutant studies
1066	Baseline studies

^*^This total includes 9443 experiments manually evaluated by GXD curators. A linear support vector classifier, a *machine* learning algorithm, was used to predict that a further 3985 microarray experiments were outside GXD’s scope. Manual evaluation of a subset of the predictions suggests that few, if any, relevant experiments have been missed.

The second step in developing the metadata index was to evaluate the appropriateness of each experiment for inclusion in the index. We chose experiments that are consistent with GXD’s scope as defined above, i.e. experiments that examine endogenous gene expression in tissues from WT and mutant mice, as well as studies examining expression differences within and between species. If all samples in an experiment are within GXD’s scope, then the experiment is included in the index. If some samples in an experiment are within GXD’s scope but others are not, then the experiment is included in the index when its GXD-consistent samples were used for a comparison within scope (e.g. tissue vs. tissue, age vs. age, WT vs. mutant). In those instances the metadata of the consistent samples will be indexed, while that of the inconsistent (e.g. the transgenic or treated) samples will not be, although a notation is made of why their metadata was not annotated. Since experiments are commonly designed to examine more than one parameter, if we discarded all experiments that contained some samples that we could not annotate, we would lose many experiments that are within the scope of the index.

Experiment titles and abstracts are often insufficient to accurately evaluate an experiment’s scope because they frequently describe the accompanying publication, rather than the experiment itself. In such cases sample information in the repository or publication is consulted. Of the 16 550 experiments evaluated to date, 3109 of them have been determined to be within our scope (Table 2). The majority of studies (12 565 of 16 550) were evaluated manually by the curators. The remaining studies (3985) were deemed to be outside GXD’s scope by a linear support vector classifier, a standard *machine* learning model, implemented in scikit-learn and whose features were word frequencies in experiment titles, abstracts and experimental factor keywords from ArrayExpress (12,13). The classifier was trained on 5600 previously evaluated experiments. When a subset of its predictions were manually evaluated, it was found to have a negative predictive value of 97%, meaning 97% of its ‘not in GXD’s scope’ predictions were correct. This freed curators from the manual evaluation of these 3985 experiments.

The third step in developing the metadata index was annotating the sample and experiment attributes of these experiments using standardized vocabularies and ontologies.

### Sample attributes

#### Anatomical structure

We use the Mouse Developmental Anatomy Ontology (14,15; http://www.obofoundry.org/ontology/emapa.html) to annotate tissues. This ontology is extensive, containing Theiler stage-specific anatomical terms hierarchically organized from tissue to tissue substructure. Use of this ontology ensures that when users search for the tissues of most interest to them, the searches will return studies that examine expression in those tissues and their substructures.

**Figure 1:**
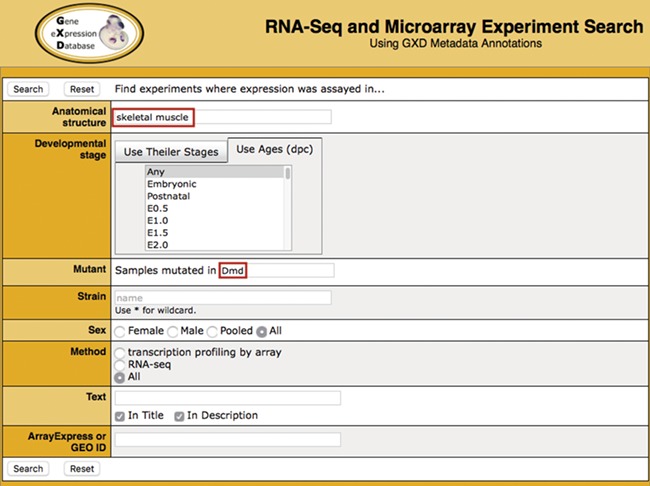
Search. Illustrated is a search for experiments studying gene expression in the skeletal muscle of *dystrophin* (*Dmd*) mutants. It takes advantage of two of the curated sample attribute fields: anatomical structure (‘skeletal muscle’) and mutant gene (‘*Dmd*’). Additional curated fields available for searching are developmental stage, strain and sex. Users can also do free text searching of experiment titles and descriptions, as well as search by ArrayExpress or GEO id.

**Figure 2:**
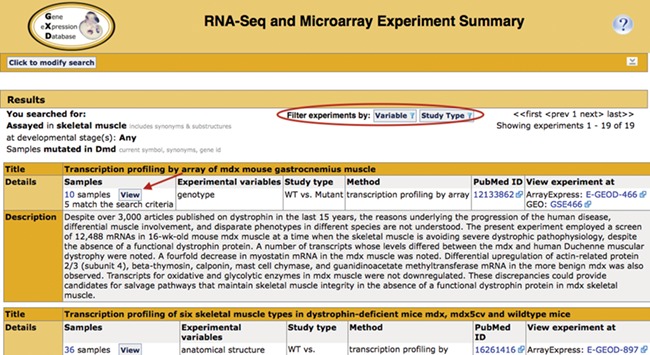
Search return. Pictured is the return for the search in Figure 1. Filters that allow for further refinement of the return are circled. The red arrow indicates the button to access the pop-up sample table (Figure 3; discussed below). The display also includes the annotated experimental variable(s) and study type, as well as link-outs to the data at ArrayExpress and GEO and the publication at PubMed.

#### Age and developmental stage

The ages provided by the data submitters are converted into standardized age terms in GXD. However, since embryos may develop at different rates, the samples are also mapped to the appropriate Theiler stage of the anatomy ontology. Theiler stages are defined by the appearance of specific developmental features (16), thus enabling the grouping of embryos by developmental features.

#### Mutant

Since different alleles of the same gene may have different phenotypes and expression patterns, GXD curators expend considerable effort to ensure that expression data from mutant mice are associated with the correct alleles. To accurately identify mutants, it is generally necessary to consult the accompanying publication. This means that the GXD sample annotations include information in addition to that included in the original repository submission. MGI maintains a complete catalog of phenotypic mutations in the laboratory mouse, each with a unique accession id.

*Strain*. Annotations follow the international guidelines for strain nomenclature (http://www.informatics.jax.org/mgihome/nomen/strains.shtml). This ensures that all specimens are entered in a consistent fashion.

*Sex*. The controlled vocabulary includes male, female, pooled and not specified.

*Species*. Non-mouse samples are indexed using the genus and species name (although the common name is used for display on the web). GXD provides no annotation of other metadata fields for non-mouse samples.

### Experiment attributes

*Study type*. The studies are grouped into two types: ‘baseline’ and ‘WT vs. mutant.’ Baseline studies examine expression in WT mice. WT vs. mutant studies are those that compare WT and mutant gene expression. The experiment study type is determined by the samples that are within GXD’s scope (i.e. those whose metadata was annotated). This means there are some instances where an experiment would be annotated to a different study type if, e.g. the transgenic or treated samples were annotated.

#### Experimental variables.

The curators also record the key experimental variables of each study using a controlled vocabulary. These variables include age, anatomical structure, developmental stage, genotype (for studies involving mutants), species, mouse species, mouse strain, pregnancy, sex and time of day (for studies involving circadian rhythms).

Currently, the RNA-seq and microarray index has metadata annotations for 3109 studies (Table 2). Two thirds of these studies are WT vs. mutant studies and one third are baseline.

**Figure 3:**
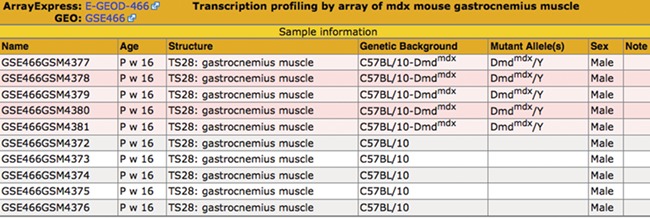
Sample table. The sample information is displayed in a pop-up table that can be accessed by using the View button (Figure 2). Samples that match the search criteria (Figure 1) are highlighted in pink. The matching samples are annotated to tissues that are ontological children of the search term skeletal muscle and carry mutations of the *dystrophin* (*Dmd*) gene.

## User Interface

### Search form

The Search Form (Fig. 1) makes it possible to query all indexed experiments using standardized sample metadata fields: anatomical structure, developmental (Theiler) stage, age, mutated gene (using either current symbol, name or synonyms), strain and sex. In addition, a free text search of experiment title and description is provided. Users can also search using the ArrayExpress or GEO id or experiment type.

### Search results summary

The Summary (Fig. 2) displays the experiments that match the search criteria, together with their standardized metadata and description. Link outs to ArrayExpress and GEO provide access to the expression data. Link outs to PubMed provide access to the corresponding publications if available. The publication field is also curated by GXD and, therefore, often includes PubMed ids that are not associated with the repository entry. The curated experiment fields (study type and experimental variables) can be used as filter values to further refine the list of returned studies.

### Sample table

The samples and their metadata are displayed in the Sample Table (Fig. 3). Samples that match the search criteria are highlighted. The illustrated search demonstrates the benefit of using a hierarchical ontology to annotate the samples—the matching samples are annotated to ‘gastrocnemius muscle,’ a substructure of the search term, ‘skeletal muscle.’

## Future Work

Analysis has begun for developing the means to directly download the metadata for experiments submitted to GEO, allowing incorporation of the more recently deposited GEO experiments into the GXD index. We also plan to incorporate the Cell Type Ontology (CL; 17,18) into our system to enable the metadata annotation of cell-type specific experiments.
